# Activated Protein C (APC) and 3K3A-APC-Induced Regression of Choroidal Neovascularization (CNV) Is Accompanied by Vascular Endothelial Growth Factor (VEGF) Reduction

**DOI:** 10.3390/biom11030358

**Published:** 2021-02-26

**Authors:** Tami Livnat, Yehonatan Weinberger, José A. Fernández, Alaa Bashir, Gil Ben-David, Dahlia Palevski, Sarina Levy-Mendelovich, Gili Kenet, Ivan Budnik, Yael Nisgav, John H. Griffin, Dov Weinberger

**Affiliations:** 1 Rabin Medical Center, Ophthalmology Department and Laboratory of Eye Research Felsenstein Medical Research Center, Petah-Tikva 49100, Israel; yoniwein@gmail.com (Y.W.); bashir.ala@gmail.com (A.B.); gil8000@gmail.com (G.B.-D.); dahlia.palevski@gmail.com (D.P.); ynisgav@gmail.com (Y.N.); dovweinb@gmail.com (D.W.); 2 Sackler Faculty of Medicine, Tel Aviv University, Tel-Aviv 69978, Israel; levysarina@gmail.com (S.L.-M.); Gili.Kenet@sheba.health.gov.il (G.K.); 3 Sheba Medical Center, The Amalia Biron Thrombosis Research Institute, Tel-Hashomer 52621, Israel; 4 Department of Molecular Medicine, The Scripps Research Institute, La Jolla, CA 92037, USA; jfernand@scripps.edu (J.A.F.); jgriffin@scripps.edu (J.H.G.); 5 Department of Pathophysiology, Sechenov First Moscow State Medical University (Sechenov University), 119019 Moscow, Russia; budnik.ivan@gmail.com

**Keywords:** activated protein C (APC), choroidal neovascularization (CNV), vascular endothelial growth factor (VEGF), Tie2, retina, mouse model

## Abstract

The activated protein C (APC) ability to inhibit choroidal neovascularization (CNV) growth and leakage was recently shown in a murine model. A modified APC, 3K3A-APC, was designed to reduce anticoagulant activity while maintaining full cytoprotective properties, thus diminishing bleeding risk. We aimed to study the ability of 3K3A-APC to induce regression of CNV and evaluate vascular endothelial growth factor (VEGF) role in APC’s activities in the retina. CNV was induced by laser photocoagulation on C57BL/6J mice. APC and 3K3A-APC were injected intravitreally after verification of CNV presence. CNV volume and vascular penetration were evaluated on retinal pigmented epithelium (RPE)-choroid flatmount by fluorescein isothiocyanate (FITC)-dextran imaging. VEGF levels were measured using immunofluorescence anti-VEGF staining. We found that 3K3A-APC induced regression of pre-existing CNV. VEGF levels, measured in the CNV lesion sites, significantly decreased upon APC and 3K3A-APC treatment. Reduction in VEGF was sustained 14 days post a single APC injection. As 3K3A-APC retained APCs’ activities, we conclude that the anticoagulant properties of APC are not mandatory for APC activities in the retina and that VEGF reduction may contribute to the protective effects of APC and 3K3A-APC. Our results highlight the potential use of 3K3A-APC as a novel treatment for CNV and other ocular pathologies.

## 1. Introduction

Choroidal neovascularization (CNV) is the pathological growth of neovascular blood vessels from the choroid underlying the retinal pigmented epithelium (RPE) towards the sensory retina. Vascular endothelial growth factor (VEGF) is the main protein that contributes to the stimulation of CNV and the breakdown of the outer blood–retina barrier (oBRB) [1,2]. Although nearly all patients with CNV are treated with intravitreal administration of drugs targeting VEGF, there remains a large unmet need for additional and alternative therapies to address the limited visual outcomes and the treatment burden with anti-VEGF therapy [3].

Activated protein C (APC) is a plasma protease possessing two distinct functions: (1) anticoagulant properties mediated by proteolysis of coagulation factors Va and VIIIa, and (2) cytoprotective effects including anti-apoptotic effects, anti-inflammatory effects, neuroprotective effects, and endothelial barrier stabilization [4]. A recombinant APC (drotrecogin alfa) was approved for adult severe sepsis treatment; however, therapy was complicated by bleeding events that were considered drug-related side effects [5]. A modified APC, 3K3A-APC, was designed to possess significantly reduced anticoagulant activity (<10%) while maintaining full cytoprotective properties and thus diminishing the risk of bleeding [6].

In a recently published study by our group [7], we used the laser-induced CNV mouse model and found that intraocular injection of APC significantly inhibited newly formed CNV. Moreover, we demonstrated that APC reduced leakage from pre-existing CNV, and its protective activities were partially mediated via the Tie2 tyrosine kinase receptor.

Tie2 is predominantly expressed by the endothelium, playing important roles in the regulation of endothelial permeability, integrity of barrier function, and angiogenesis [8,9,10]. Until recently, the angiopoietins (Ang) were the only known ligands for Tie2, with Ang1 and Ang2 being the most abundant and best studied [8,9,10]. Nonetheless, Minhas et al. showed that APC is a direct agonist of Tie2; its binding to Tie2 mimics Ang1 binding and induces rapid activation of the receptor and initiates downstream signaling pathways [11]. The angiopoietin–Tie2 system has been identified as an important axis for clinical drug development for retinal diseases, as it plays a complementary role alongside VEGF. Thus, significant efforts are devoted to drug development that combines VEGF and Tie2 pathways and defines an ideal treatment for retinal diseases [8,9,10,12].

In the current study, we aimed to assess the mode of APC’s protective activities in the retina via VEGF. Moreover, as 3K3A-APC has reduced anticoagulant properties and therefore holds higher potential as an intraocular treatment, we aimed to study whether 3K3A-APC preserves APC’s ability to regress pre-existing CNV and reduce VEGF expression.

## 2. Materials and Methods

### 2.1. In Vivo Laser-Induced CNV Animal Model

The study included 132 eight-week-old male C57BL/6J mice weighing 19 to 25 g that were purchased from Envigo RMS, Rechovot, Israel. All animal experiments were performed according to the Association for Research in Vision and Ophthalmology (ARVO) statement’s guidelines for the Use of Animals in Ophthalmic and Visual Research and the approval of the Institutional Animal Care and Use Committee at Rabin Medical Center. CNV was induced based on Weinberger et al. [13] as previously described [7]. Briefly, diode laser indirect ophthalmoscope (Iris Medical Oculight SLX System©, Iridex, Mountain View, CA, USA) was used with a laser power of 350 mW for a duration of 100 msec and a condensing lens of 90 diopters. Three laser applications were applied to the right eyes at a distance of 1 to 2 optic disc diameters around the optic nerve. Disruption of the Bruch’s membrane was identified by the appearance of a white bubble at the site of photocoagulation.

Human APC (Haemathologic Technologies Inc., Essex Junction, VT, USA) or murine recombinant 3K3A-APC (KKK192-194AAA) were prepared as previously described [6,7].

APC and 3K3A-APC were diluted in saline (1 µg/µL/eye) and were injected intravitreally (ITV) under an operating microscope (Zeiss Opmi 6S Microscope; Carl Zeiss Microscopy GmbH, Oberkochen, Germany) using a microsyringe (33 gauge; Hamilton, Reno, NV, USA). Mice injected with saline, with or without laser application, served as controls. Animals were anesthetized with intraperitoneal (IP) injection of ketamine 100 mg/kg and xylazine 10 mg/kg. For all animal experiments, animal allocation to treatments was randomized, and each experiment was repeated 2–3 times.

### 2.2. Vascular Imaging and Flatmount Immunostaining

On days 5 to 7 post CNV induction, mice were injected ITV with either 1 µg/µL APC or 3K3A-APC or with saline. Mice without laser application were injected with saline and served as additional control. Seven to nine mice per group were anesthetized, and 0.1 mL of 25 mg/mL fluorescein isothiocyanate (FITC) dextran conjugate (MW 500k, Sigma Aldrich, Rehovot, Israel) was injected into the left ventricle of the mouse heart. Five minutes later, the mice were sacrificed, and a flatmount specimen of RPE-choroid was separated from the eyecup and flattened on slides. Flatmount specimens were fixed in 4% para formaldehyde (PFA) for 10 min. The slides were covered with anti-fade reagent (Invitrogen, Carlsbad, CA, USA). For anti-VEGF immunostaining, flat fixed RPE-choroid slides were incubated in phosphate-buffered saline (PBS)-Triton X100 0.5% solution at 4 °C overnight and later blocked for 2 h at room temperature (RT) in 5% normal donkey serum (NDS; Sigma Aldrich, Israel). Slides were incubated with rabbit anti-mouse VEGF antibody (1:200; Abcam, Cambridge, UK) at 4 °C overnight, followed by incubation with Alexa Fluor 568 conjugated goat anti-rabbit IgG (1:100; Invitrogen, Camarillo, CA, USA). Slides were covered with anti-fade. A specimen incubated with non-immune serum was used as staining control. The choroid-RPE specimens were scanned from the RPE towards the choriocapillaris layer. Images of 3-dimensional (3D) projections were captured as previously described [7]. Using the Leica TCS SP8 confocal microscope (Leica Biosystems, Nussloch, Germany), Z-stack images were taken under identical conditions. The volume and the depth of VEGF and FITC dextran (CNV) staining were measured using the Imaris x64 7.1.1 software (Oxford Instruments, High Wycombe, UK).

### 2.3. Cryosections Histology and Immunofluorescence Staining

On days 1–30 post APC treatment, mice were sacrificed (*n* = 4 mice per group, a total of 32 mice). Eyes were removed, punched with a 30 g needle, and fixed in 4% PFA for 2 h at RT. Eyes were washed with increasing concentrations of sucrose in PBS and gradually incubated with a final concentration of 30% sucrose overnight at 4 °C. Eyes were then embedded in Tissue-Tek Optimal cutting temperature (O.C.T) compound (Sakura Finetek, Tokyo, Japan) on dry ice and kept at −80 °C. Serial sections of 10 μm thickness were cut using a cryostat (Leica Biosystems, Nussloch, Germany). Sequential cryosections of every fifth section of each eye were stained with anti-VEGF antibodies as follows: cryosections were blocked with 10% NDS for 1 h RT and incubated with rabbit anti-mouse VEGF antibody at 4 °C overnight (1:400) followed by incubation with Alexa Fluor 568 conjugated goat anti-rabbit IgG (1:100). Finally, nuclei were counterstained with 4′,6-diamidino-2-phenylindole, (DAPI) Nucblue fixed cell stain, Molecular Probes, Eugene, OR, USA). Images were captured under the same settings using a fluorescence microscope (Axio Imager.Z2, Carl Zeiss Microscopy GmbH, Jena, Germany). VEGF staining from lesion sites was scored from 0–3 (light to heavy staining, respectively) by 2 masked observers for a total of 5 slides per mouse and 20 photos per group. Sequential cryosections with comparing regions were stained for hematoxylin and eosin stain (H&E) (ScyTek Laboratories Inc., Logan, UT, USA). Images were captured using a fluorescence microscope (Axio Imager).

### 2.4. Fluorescein Angiography (FA)

After the mice were anesthetized, their pupils were dilated using tropicamide 0.5%, and 0.1 mL 2.5% fluorescein sodium (Novartis, Basel, Switzerland) was injected IP. Sequential real-time photos were captured during the early phase (during the first minute from fluorescein injection) and the late phase (every minute between 2 to 5 min following fluorescein injection). Color fundus photographs and fluorescein angiography (FA) images were taken using the Optos California UWF imaging system (Optos Inc., Southborough, MA, USA). Two masked retina specialists evaluated the fluorescein angiograms and sorted each laser spot as “leakage”, hyperfluorescent lesion with blurred margins increasing in size over time.

### 2.5. Statistical Analysis

Statistical analysis was performed using SPSS version 23 (IBM Corp., Armonk, NY, USA). The data were presented as mean ± standard deviation (SD). The effect of APC on CNV and VEGF volume and depth was evaluated using one-way ANOVA followed by Tukey post hoc test. The effect of APC on mean scoring of VEGF staining at four time points was evaluated by unpaired two-tailed Student’s *t* test (the resulting *p* values were adjusted for the number of time points using Sidak’s correction). Differences were considered statistically significant if the *p* value was less than 0.05.

## 3. Results

### 3.1. APC-Induced Regression of CNV and VEGF Reduction; Flatmount Specimens’ Evaluation

**Figure 1:**
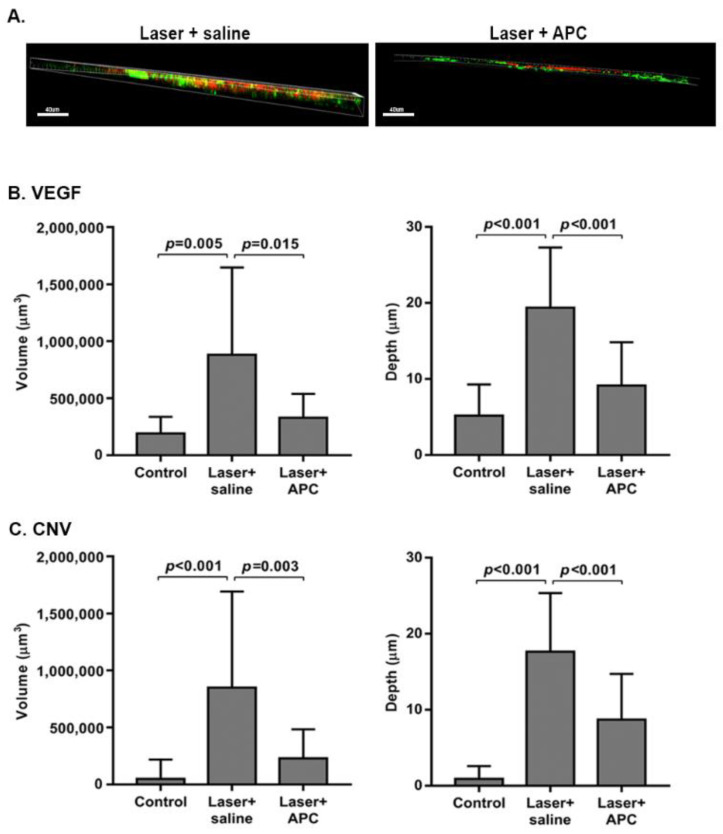
**APC treatment induces a reduction in VEGF levels and regression of CNV.** Six days post-laser application, CNV was verified by FA, and mice were treated with 1 µg/µL/mouse APC or saline. Mice without laser application that were injected with saline served as a control. Three days later, CNV was stained using FITC-dextran perfusion, and flat RPE-choroid specimens were isolated and stained with anti-VEGF antibodies. Panel (**A**) shows a Z-plane immunofluorescent image of RPE-choroid flatmounts taken from eyes subjected to laser and treated with APC or saline (scale bar represents 40 µm; red, VEGF; green, CNV). Quantifications of VEGF and CNV volume (µm^3^) and depth (µm) were measured using Imaris and are depicted in panels (**B**,**C**), respectively. The data are presented as mean ± SD (7–9 mice per group) and were analyzed using one-way ANOVA followed by Tukey post hoc test.

Leaky pathological blood vessels are already present in the retina in most patients with CNV when diagnosed. Thus, to mimic the clinical situation, we initially induced CNV growth by laser photocoagulation, and only mice with confirmed leakage from CNV were subjected to further evaluation. After FA verified CNV presence, mice were injected ITV with 1 µg/µL APC or saline (concentration was depicted based on dose-dependent analysis previously performed) [7]. Three days post-APC treatment, the expression of VEGF was examined while it was highly expressed [14,15]. Figure 1A shows representative Z-plane color images of VEGF (red) and CNV (green) in eyes subjected to laser treated with saline or APC. Quantitative measurements of volume and depth showed a significant reduction in the mean volume of VEGF in the APC-treated mice compared to untreated mice (328,457 ± 211,489 µm^3^ and 881,200 ± 765,650 µm^3^ (*p* = 0.015), respectively). Moreover, the VEGF staining, which was demonstrated throughout the entire depth of choroid-Bruch’s membrane-RPE depth in the saline-treated eyes (19.4 ± 7.9 µm), was restricted to the RPE region (9.1 ± 5.7 µm depth (*p* < 0.001) in the APC-treated eyes (Figure 1B). Along with VEGF reduction, APC treatment induced a statistically significant reduction in CNV volume, from a mean of 849,173 ± 842,793 µm^3^ in saline-treated eyes to 229,219 ± 254,137µm^3^ in APC treated eyes (*p* = 0.003) and a 50% reduction in the depth of CNV invasion (*p* < 0.001) (Figure 1C).

### 3.2. APC Time-Dependently Reduced VEGF Levels at CNV Lesion Sites

**Figure 2:**
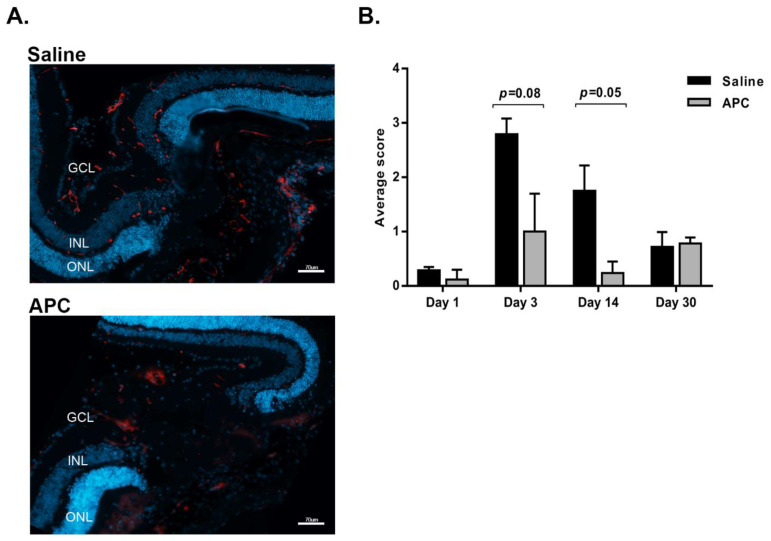
**Longitudinal effect of APC on VEGF levels in CNV lesion sites.** Immediately following laser applications, APC (1 µg/µL/mouse) or saline were injected intravitreally (ITV), and 1, 3, 14, and 30 days later, mice were sacrificed (*n* = 4 mice per group). Sequential cryosections were stained with anti-VEGF antibodies (red), and cell nuclei were stained with DAPI (blue). (**A**) Representative histological sections image of laser lesion sites from mice treated with APC or saline, taken 3 days post treatment. GCL, ganglion cells layer; INL, inner nuclear layer; ONL, outer nuclear layer. Scale bar, 70 µm. Panel (**B**) summarizes the mean scoring of VEGF staining in laser lesion sites. Images of every fifth slide of each eye were captured digitally under the same settings, and VEGF staining was scored from 0–3 (light to heavy staining, respectively) by 2 masked observers. Twenty images per group were used for analysis. The data are presented as mean ± SD and were analyzed using unpaired two-tailed Student’s *t* test (the resulting *p* values were adjusted for four time points using Sidak’s correction).

Our previously published data showed that CNV growth was suppressed even 2 weeks following only a single dose injection of APC [7]. We now evaluated VEGF levels in cryosections 1, 3, 14, and 30 days post-APC (1 µg/µL) treatment. A representative image of laser lesion sites (Figure 2A) taken 3 days post laser photocoagulation demonstrates that VEGF (red) staining was less prominent at the lesion site of APC-treated eyes in comparison to saline-treated eyes. Figure 2B summarizes the mean scoring of VEGF staining 1–30 days post laser application. As expected, VEGF levels increased dramatically after laser application, with a peak at 3 days post laser. A single ITV injection of APC induced a statistically significant longitudinal reduction in VEGF levels in the lesion sites 3 days after treatment (*p* = 0.08), an effect lasting 14 days post-treatment (*p* = 0.05). Thirty days post-treatment, VEGF levels decreased with no difference between treated and untreated eyes.

### 3.3. 3K3A-APC Induces Regression of CNV

**Figure 3:**
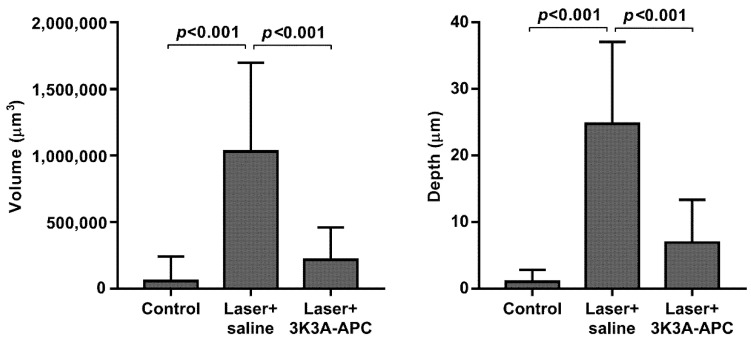
**3K3A-APC induced regression of CNV.** Six days post laser, CNV presence was confirmed by FA, and mice were injected ITV with 1 µg/µL 3K3A-APC or with saline. Mice without laser application were injected with saline and served as a control. Seven days later, blood vessels were stained using FITC-dextran perfusion, choroidal flatmounts were isolated, and 3D confocal images were scanned. Quantification of the depth (µm) and the volume (µm ^3^) of the CNV was measured using Imaris. The data are presented as mean ± SD (7–9 mice per group) and were analyzed using one-way ANOVA followed by Tukey post hoc test.

Since retinal hemorrhages can cause severe vision impairment, particularly when associated with CNV, we tested whether the APC variant 3K3A-APC, with reduced anticoagulant activity, would retain APCs’ activity and reduce CNV growth and penetration toward the sensory retina. FA verified CNV presence, and mice were injected with 1 µg/µL 3K3A-APC or with saline. A quantitative assessment of CNV volume and depth performed 7 days later is shown in Figure 3. CNV volume was minimal at baseline without laser application (control, 57,050 ± 182,989 µm^3^). Laser applications led to an increase up to 1,030,083 ± 667,833 µm^3^, while 3K3A-APC treatment reduced CNV volume to 217,000 ± 241,423 µm^3^ (*p* < 0.001). Laser applications led to an increase in blood vessel penetration depth from 1.1 ± 1.8 to 24.8 ± 12.3 µm (*p* < 0.001), while 3K3A-APC treatment reduced vascular invasion depth to 6.9 ± 6.4 µm (*p* < 0.001).

### 3.4. 3K3A-APC Reduces VEGF Levels at CNV Lesion Sites

At the next stage, we tested whether 3K3A-APC retained APC’s ability to reduce VEGF levels.

**Figure 4:**
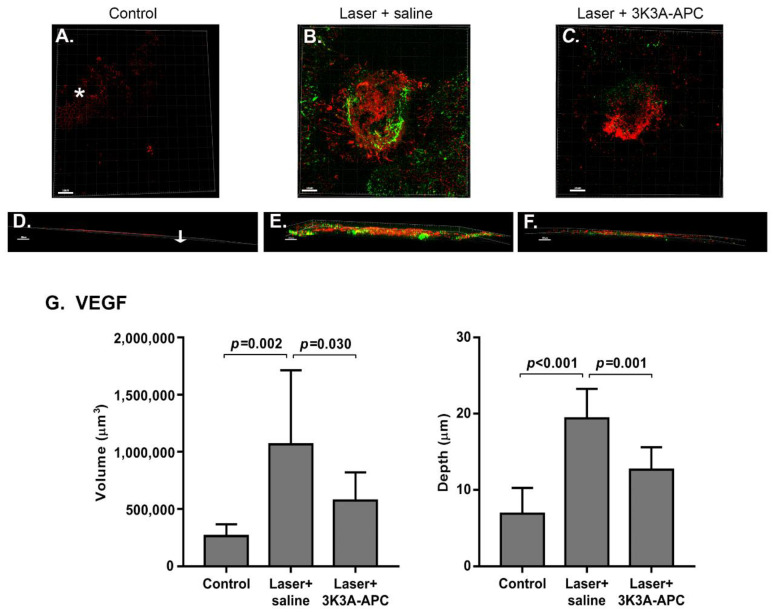
**3K3A-APC reduced VEGF levels.** Immediately following laser applications, mice were treated with 1 µg/µL 3K3A-APC or saline. Mice without laser applications served as a control. Three days later, CNV was stained using fluorescein isothiocyanate (FITC)-dextran perfusion, and flat retinal pigmented epithelium (RPE)-choroid specimens were isolated and stained with anti-VEGF antibodies. An immunofluorescent image of RPE-choroid flatmounts average laser-induced CNV spot size, upper view (**A**–**C**), and Z-plane (**D**–**F**). Scale bar represents 50 µm and 30 µm for upper view and Z-plane, respectively. The arrow shows the Z-plane (depth), and the asterisk indicates VEGF hexagonal staining of the RPE membrane. Red, VEGF; green, CNV. (**G**) A quantification of the volume (µm^3^) and the depth (µm) of VEGF in eyes without laser application (control) and eyes subjected to laser and treated with either saline (laser + saline) or with 3K3A-APC (laser + 3K3A-APC). The data are presented as mean ± SD (7–9 mice per group) and were analyzed using one-way ANOVA followed by Tukey post hoc test.

Mice subjected to laser were treated with 1 µg/µL 3K3A-APC or saline and sacrificed 3 days later. Figure 4 shows representative upper view (A–C) and Z-plane (D–F) color images of RPE-choroid flatmounts performed 3 days post-treatment. In eyes not exposed to laser applications (control eyes), only slight staining of VEGF was detected, with a typical hexagonal honeycomb pattern (pointed by the asterisk) corresponding to the edge of the RPE and without any vascular staining (Figure 4A). Z-axis view shows that VEGF was restricted to the RPE edge, and no CNV was detected across the entire depth of the scanned cube (Figure 4D). In eyes with CNV treated with saline, VEGF and vascular staining were demonstrated throughout the entire depth of choroid-Bruch’s membrane-RPE with a strong classic staining appearance of the CNV lesion in the upper side of the RPE (Figure 4B,E). In eyes treated with 3K3A-APC, the upper view indicates a dramatic reduction in blood vessels reaching the RPE layer with a significant reduction in VEGF expression (Figure 4C,F). Quantitative assessment of VEGF volume and depth in the entire specimens is shown in Figure 4G. The total volume of VEGF in control eyes was 264,000 ± 104,170 µm^3^, which increased upon laser to 1,066,286 ± 648,214 µm^3^ (*p* = 0.002) and reduced by 3K3A-APC treatment to 574,583 ± 245,810 µm^3^ (*p* = 0.030).

Furthermore, 3K3A-APC treatment resulted in a reduced vascular invasion depth from 19.4 ± 3.9 µm in laser-treated eyes to 12.7 ± 2.9 µm in the 3K3A-APC treated eyes (*p* < 0.001).

## 4. Discussion

In the current study, we elaborate on the data regarding APC use as a potential treatment for CNV. We previously demonstrated that APC has the ability to inhibit the growth of newly formed CNV, inhibit pathological leakage from CNV, and tighten the oBRB. Moreover, we showed that APC’s protective activities in the retina were mediated by the Tie2 receptor [7].

As APC is a natural coagulation inhibitor, bleeding risk may be a major issue. Clinical studies showed that APC therapy increased the risk for serious bleeding events in adult patients with severe sepsis [16,17]. 3K3A-APC is a recombinant engineered variant of APC (three Lys residues replace three Ala residues) with markedly reduced anticoagulant activity. Although the replacement of the three residues in the 3K3A-APC variant reduces APC’s interactions with its substrate, clotting factor Va, it does not affect APC’s interactions with its cell receptors, including binding to endothelial protein C receptor and activating protease-activated receptors (PAR) 1 and 3 [4,6,18]. Thus, both wild-type APC and 3K3A-APC have anti-apoptotic, anti-inflammatory, neuroprotective, cytoprotective, and endothelial barrier stabilization properties [4,18].

To evaluate the potential clinical use of APC, we attempted to simulate the clinical setting, wherein pathological leakage is already present in most of the patients diagnosed with CNV. We applied treatment just after confirming the presence of leakage from CNV by FA and demonstrated that, similarly to APC, 3K3A-APC induces CNV regression. Our results suggest that the anticoagulant properties of APC are not mandatory for its beneficial effects on the retina. Blood vessels involved in the pathogenesis of CNV are immature, lack structural integrity, and leak fluid, leading to hemorrhage and exudates accompanied by fibrosis and loss of vision [3]. Therefore, 3K3A-APC may prevent exposure of the intraocular environment to the anticoagulant properties of APC that may pose a risk for retinal hemorrhage. Thus, 3K3A-APC holds high significance for moving ahead to clinical trials.

VEGF is expressed within the normal retina even in the absence of active angiogenesis [19,20,21], working as an important neurotrophic factor crucial for survival and normal function of the retina [2]. In the aging eye, the pathologic level of VEGF released by the RPE was a detrimental factor in developing CNV and neovascular age-related macular degeneration nAMD [1,22]. The current efficacious treatments for CNV are mostly based on anti-VEGF agents, yet, suboptimal response, slow loss of efficacy, and long term concerns regarding the continuity of the neurodegenerative process restricted their full success [1,2,23]. Using the laser-induced CNV mouse model, we previously demonstrated that APC treatment reduced CNV to a similar extent as bevacizumab [7,13].

In the current study, we demonstrate that the enhanced expression of VEGF, detected in the mice eyes with CNV, was significantly reduced upon APC or 3K3A-APC treatment. Moreover, VEGF is known to be secreted in a polarized manner from the basolateral aspect of the RPE toward the choriocapillaris layer [24]. We scanned the VEGF gradient from the RPE toward the choriocapillaris and found that VEGF expression was detected in all depths of RPE-choroid specimens of eyes with CNV that were treated with saline; however, treatment with APC and 3K3A-APC reversed the expanded expression of VEGF and restricted it to the RPE edge. Interestingly, the APC-induced reduction of VEGF expression in CNV lesions sites was sustained up to 2 weeks post single injection. These results support our previously reported findings that showed a reduction in CNV two weeks after a single dose of APC treatment [7].

In laser-induced CNV, VEGF elevation enhances the production of intercellular adhesion molecule (ICAM), which is expressed in RPE and on vascular endothelial cell surfaces and is an important component of cell-to-cell interactions [25]. Chemokines and cytokines attract macrophages via chemokine receptors such as C-C chemokine receptor type 2 (CCR2) to the CNV site and promote their activation. Accordingly, infiltrating macrophages secrete inflammatory cytokines which amplify VEGF-secreting macrophage recruitment to the CNV site, further augmenting local VEGF production and CNV development [15,26,27,28]. Currently, we do not have biochemical data explaining APCs’ mechanism of action in the retina. However, we hypothesize that VEGF reduction and CNV suppression, demonstrated herein, could have evolved from APCs’ pleiotropic activities. APC is known to downregulate vascular adhesion molecules such as ICAM-1 to diminish leukocyte release cytokines such as monocyte chemoattractant protein 1 (MCP-1) and reduce leukocyte adhesion and infiltration [29,30,31,32]. As macrophage depletion is associated with VEGF reduction and CNV inhibition [15,26,27,28], APCs’ activities on endothelial cells and mononuclear cells may explain our observations. APC may exert its effect, at least partially, through reduction of adhesion, infiltration, and accumulation of monocytes in the CNV site. Moreover, recent studies suggest that APC can act via inhibition of macrophage inflammasome activity [33], which is considered a novel therapy target for treating nAMD [28]. Thus, APC’s multiple target and multiple action may explain our findings regarding VEGF reduction and CNV suppression in a laser-induced model of CNV.

The ability of APC to reduce VEGF expression and to oppose VEGF-induced endothelial permeability was previously demonstrated in-vivo in a skin model [34] and a diabetic nephropathy mouse model [35]. In contrast, an elevation in VEGF levels and a proangiogenic effect of APC were demonstrated in other pathological models [36,37,38,39,40]. The angiogenic process’ complexity and the variability between vascular beds may modify the balance between pro-angiogenic and anti-angiogenic effects.

Disruption of the outer BRB is a crucial step in the pathogenesis of CNV [1]. Barrier stabilizing properties, including the RPE barrier tightening induced by APC [4,7,18,34,40], may contribute to CNV suppression. By stabilizing the RPE barrier integrity, APC may restrict choroidal endothelial cell migration through the RPE and limit CNV invasion into the sensory retina. Moreover, APC’s effects may preserve RPE polarity and thus avoid misdirection of secreted VEGF and subsequent classical subretinal neovascularization [20,21,22,24].

Currently, considerable research addresses the hypothesis that combining VEGF inhibitors with activators of the tyrosine kinase Tie2 receptor may achieve greater efficacy than anti-VEGF monotherapy. The potential advantage of combined therapy may stem from the stabilizing role of Tie2 on the vasculature with a potential reduction in vascular leakage [8,10,24].

Although the angiopoietins are critically recognized as key ligands for Tie2 receptor [8,10], Minhas et al. showed that APC is also a direct agonist for Tie2 [11]. APC binding to Tie2 initiates rapid activation of the receptor, which manifests in barrier protection of endothelial cells and ultimately leads to vascular stability. These actions of APC mimic those of Ang1 and are supported by the functional similarities between Ang1 and APC [11]. In concordance with these findings, we demonstrated that APC’s suppression and regression of CNV were mediated by the Tie-2 receptor, and blockage of the Tie2 receptor eliminated APC’s beneficial activities in the retina in-vivo and in-vitro [7].

The broad range of injuries for which APC or 3K3A-APC provide pharmacologic benefits lead to its translation to the clinic [4,18,41]. Safety and efficacy of intravitreal administration of APC were confirmed in a prospective pilot clinical trial for patients with central retinal vein occlusion (CRVO), which demonstrated an improvement in the clinical course. Moreover, the authors reported complete resolution of macular edema in five out of ten eyes following one injection of APC with no recurrence during 1 year follow-up [42].

Ocular pathologies associated with CNV (including nAMD) are characterized by the pathogenic triad consisting of vascular damage, neuronal injury, and neuroinflammation [30]. We believe that APC treatment can control and limit the vicious cycle that exacerbates the retina’s damage. Additional studies should be conducted to clarify this hypothesis.

## 5. Conclusions

The protective effects of APC and 3K3A-APC in the retina seem to be promising as potential treatment for CNV and nAMD. APC and 3K3A-APC may modulate CNV through the combination of suppressing VEGF and activating Tie2. The current treatments for CNV are based mostly on anti-VEGF agents. However, the long term unmet need for neuroprotection has not yet been established. Both APC and 3K3A-APC share well established anti-apoptotic, anti-inflammatory, endothelial barrier stabilization, and neuroprotective effects [4,18]. Thus, their therapeutic impact upon ocular diseases is beyond CNV suppression. As the neuroprotective activity of 3K3A-APC has already been translated to phase II clinical studies for acute ischemic stroke [41], we aspire that our mice model studies will lead to a human clinical proof of concept trial that will evaluate the safety and the efficacy of 3K3A-APC in patients with CNV and nAMD.

## Data Availability

Data are available from T.L. upon request.
